# The Effect of Smoking on Salivary Calcium Levels, Calcium Intake, and Bleeding on Probing in Female

**DOI:** 10.1155/2021/2221112

**Published:** 2021-12-18

**Authors:** Sri Tjahajawati, Anggun Rafisa, Endah Ayu Lestari

**Affiliations:** ^1^Oral Biology Department, Faculty of Dentistry, Universitas Padjadjaran, Bandung, Indonesia; ^2^School of Dentistry, Faculty of Dentistry, Universitas Padjadjaran, Bandung, Indonesia

## Abstract

**Introduction:**

Smoking is a bad habit that affects both systemic and oral conditions. Nicotine in cigarettes reduces estrogen production that can alter salivary calcium levels. Nicotine also causes vasoconstriction of the gingival blood vessels and decreases gingival bleeding. Low dietary calcium intake is also suspected to influence the low serum calcium levels in smokers. In this study, we evaluated the effect of smoking on salivary calcium levels, calcium intake, and BOP in women.

**Method:**

This was an analytical study using a cross-sectional approach. The subjects were 26 female smokers and 37 nonsmokers. Unstimulated saliva was collected by the spitting method. Salivary calcium levels were measured using an Atomic Absorption Spectrophotometer (AAS). The calcium intake was obtained by the Semiquantitative Food Frequency Questionnaire. BOP was measured by a gingival bleeding index by Ainamo and Bay in 1975.

**Results:**

All the basic characteristics including age, BMI, level of education, and occupation were statistically different between groups. The mean calcium level of female smokers was significantly lower than that of nonsmokers, whereas the mean BOP of female smokers was significantly higher. The total calcium intake per day of the two groups was not statistically different. The mean salivary calcium level and BOP decreased when the duration of smoking was longer. There was a positive correlation between salivary calcium level and BOP in the smokers' group.

**Conclusion:**

A low level of education may be contributing to the smoking habit of subjects in this study. Salivary calcium levels were correlated with BOP in female smokers, which might be affected by the duration of smoking.

## 1. Introduction

Smoking is no longer identical to only men but becoming commonplace among women. The number of smokers has increased recently, despite the decline in smoking prevalence [1]. A systematic review by Jafari et al. found that 13% of adult females and 15% of adolescent females were smokers. The study also discovered a high prevalence of smoking in pregnant women (21%) [2].

The dangers of smoking can affect anyone, but women face a greater risk for morbidity and mortality caused by smoking. Female smokers have a 25% higher risk of heart disease and lung cancer when compared to male smokers because women weighed and have blood vessels less than men [3]. Smoking not only causes a systemic effect but can also causes physiopathological conditions in the oral cavity [4]. The heat from burning cigarettes can directly irritate the oral mucosa, causing damage to the salivary glands, and results in decreased salivary function [5].

Saliva consists of 99% of water and various electrolytes. Calcium is one of the inorganic electrolyte components found in saliva in the form of ions which has a role in body processes, especially in the oral cavity [6]. A study by Bafghi et al. in 2015 found a significant decrease in the total average concentration of protein, calcium, lead, and zinc in smokers compared to nonsmokers [7]. On the contrary, Abed et al. in 2015 found a significant increase in salivary calcium levels in smokers [8]. High salivary calcium levels can result in rapid plaque mineralization, thus accelerating the calculus formation in the oral cavity. Calculus can increase susceptibility to periodontal disease in the form of attachment loss, pocket depth, tooth loss, and decreased alveolar bone [9].

Women who smoke cigarettes can also experience the decreased permeability of peripheral blood vessels. The effect may be related to the level of cigarette smoke inhalation and nicotine absorption. Nicotine causes vasoconstriction of peripheral blood vessels, minimizing blood supply and oxygen to the gingiva. As a result, the response of bleeding on probing in smokers is lower [10].

The nicotine contained in cigarettes can reduce estrogen levels [11, 12]. Decreased estrogen hormone results in reduced calcium absorption in the digestive tract and decreased serum calcium levels [13]. Low dietary calcium intake is also suspected to influence the low serum calcium levels in smokers. Morabia et al. found that smokers had lower calcium intake than nonsmokers [14]. Low calcium intake can, therefore, accelerate bone loss [15].

However, studies on whether calcium intake and BOP play a role in salivary calcium level changes in female smokers have not been conducted. Therefore, in this study, we evaluated the effect of smoking on salivary calcium levels, calcium intake, and BOP in women.

## 2. Methods

This study was an analytical study using a cross-sectional approach. This study was conducted after obtaining ethical clearance or feasibility of conduct from the Research Ethics Committee, Faculty of Medicine, Universitas Padjadjaran (No. 1431/UN6.KEP/EC/2019).

The study population was female smokers and nonsmokers, who lived in Jatinangor and Bandung, Indonesia. The study subjects were 26 female smokers and 37 nonsmokers. The research was conducted from October to November 2019 at Posyandu Merpati VI Dusun Sukawening Jatinangor, Posyandu Dusun Cikeruh Jatinangor, Posyandu Merpati Dusun Sukanegla Jatinangor, and RSGM Unpad Sekeloa.

We used a nonrandom consecutive sampling method by distributing questionnaires to subjects who met the research criteria within a certain period until meeting the target sample. We asked for the consent of the respondents to participate in the study. The age criteria of subjects included in this study were 18–64 years because the highest prevalence of female smokers is in the reproductive age.

Subjects were asked to complete a smoking and alcohol habit questionnaire and then divided into smokers and nonsmokers (control) groups. The female smokers' group consisted of those who had smoked at least one tobacco cigarette every day for the last one year (active smoker), while the control group consisted of subjects who had no history of smoking. Former female smokers were not included in this study since smoking cessation was found to increase the gingival blood flow and bleeding [16]. Subjects with diseases such as diabetes mellitus, Bell's palsy, Sjogren's syndrome, HIV, and tuberculosis were not included in the study. Subjects who were menstruating, pregnant, or in menopause at the time of examination, using orthodontic appliances, and had alcohol habits were also excluded from the study. History of periodontal treatment was not considered as an exclusion criterion in this study because the population was a group that did not regularly visit the dentist; thereby, periodontal treatment was not considered as a confounding variable.

Study subjects were interviewed regarding their calcium intake during the last month using the Semiquantitative Food Frequency Questionnaire (FFQ) with the help of pictures to show the portion size of each food. Unstimulated whole saliva was collected by the spitting method. The respondents were not allowed to eat, drink, and brush their teeth 60 minutes before and during saliva collection. Respondents were asked to rest in an upright position with their head down, not move their tongue and teeth, keep their lips closed, and not swallow in one minute. Respondents were instructed to spit saliva into a centrifuge tube. This step was repeated five times for a total of five minutes to record the total salivary volume. Salivary calcium level was measured by using the Atomic Absorption Spectrophotometer (AAS) AAnalyst 400 (Perkin Elmer, Waltham, MA, USA) at the central laboratory of Universitas Padjadjaran.

The BOP was examined using the gingival bleeding index (GBI) by Ainamo and Bay. This index is the simplification of gingival bleeding (GI) index which describes the absence or presence of bleeding after gentle probing is recorded [17]. This study focused on gingival inflammation regardless of the severity of the inflammation; therefore, examination using GBI by Ainamo Bay was considered suitable for this study. This index is also easy to interpret and evaluate the condition of the gingiva. Compared to the GI index by Löe and Silness (1963), this index was also correlated significantly [18, 19].

Absolute counts and percentages were used to describe categorical variables such as age, body mass index (BMI), level of education, and occupation. Descriptive statistics of continuous variables including mean and standard deviation were measured. A Mann–Whitney *U* test was applied to compare the difference between salivary calcium levels, total calcium intake, and BOP. The Kruskal–Wallis test was used to compare salivary calcium levels, total calcium intake, and BOP based on smoking characteristics. The correlation of salivary calcium levels with total calcium intake and BOP were measured using partial correlation after adjusting the age, BMI, and level of education. The results were considered significant if *p* values < 0.05. The research data were processed with Microsoft Excel 2016 (Microsoft Corp, Version 16.0. Redmond, WA), IBM SPSS Statistics (IBM Corp, Version 26.0. Armonk, NY), and displayed in a tabular form.

## 3. Results

The basic characteristics of study subjects are shown in Table 1. All the basic characteristics including age, BMI, level of education, and occupation were statistically different between female smokers and nonsmokers. The largest proportion of female smokers in this study was in the middle-aged group (80.8%). The majority of female smokers had a BMI above 25 (76.9%) which can be identified as overweight or obese. Most female smokers received education only up to the primary level (80.8%) and were housewives (76.9%), while the control subjects had more varied occupations.

Both groups of study subjects had significant differences in salivary calcium levels and BOP (Table 2). The mean calcium level of female smokers (1.16 mmol/L) was lower than that of nonsmokers (1.8 mmol/L), whereas the mean BOP of female smokers was higher. The total calcium intake per day of the two groups was not statistically different.

Based on the duration of smoking, there was a significant difference between salivary calcium level and BOP (Table 3). The mean salivary calcium level and BOP decreased when the duration of smoking was longer. Based on the types of tobacco products, only BOP showed a statistically significant difference. The mean BOP was the lowest in the subject who used filtered kretek cigarettes. The three variables measured in the study did not show a significant difference based on the number of smoked tobacco consumption/day.

Table 4 shows the correlation of salivary calcium levels with total calcium intake and BOP after adjusting age, BMI, and level of education of the two groups. Salivary calcium levels were positively correlated with BOP in the female smokers group.

## 4. Discussion

### 4.1. Basic Characteristics

The highest prevalence rates of female smokers in this study occurred in the middle-aged group. Ng et al. also found the highest prevalence of smoking in women in developing countries was at age 20–49 years [1]. Smoking habits in women of reproductive age affect fertility, pregnancy conditions, and fetal health [20, 21]. Some studies found that this risk increased with higher nicotine doses and longer duration of smoking [21, 22].

Our finding shows the BMI of both groups was statistically significant and most female smokers were overweight. The relationship between smoking and BMI in previous studies showed conflicting results. Some studies have found no impact of smoking on obesity in female smokers [23, 24]. Other studies have found that current female smokers have a lower BMI compared to nonsmokers [25] and heavy smokers have a higher risk of obesity [26].

A study by Chéruel et al. stated that smokers had lower taste sensitivity compared to nonsmokers [27]. Taste sensitivity is associated with food preferences and eating habits. Decreased taste sensitivity leads to higher calorie, salt, and fat intake, causing an increase in BMI [28]. Lower taste sensitivity is also associated with higher blood pressure in female smokers [29].

A higher prevalence of smoking habits was associated with a lower level of education [30, 31]. A low level of education contributes to a lack of understanding about the dangers of smoking, thereby increasing health-endangering behavior and lifestyle [32]. Low levels of education are also associated with a poorer-quality diet [33, 34]. This might explain why the majority of smoking subjects have a higher BMI. The majority of female smokers work as housewives, in accordance with the RISKESDAS (Basic Health Research) data in Indonesia in 2013, which states that the highest percentage of smokers is 44.5% in the nonworking population group, including housewives [35].

### 4.2. Salivary Calcium Levels

Salivary calcium levels in female smokers tended to decrease compared to those in nonsmokers with normal salivary calcium levels of 1.32 ± 0.24 mmol/L. These results were in line with several studies. Bafghi et al. stated that salivary calcium levels in smokers were lower, although not significant compared to those in nonsmokers [7]. Similar to the study of Zuabi et al. the ion compositions of saliva (Ca^2+^, Mg^2+^, and Na^+^) in smokers has significantly lower differences [36]. In contrast to the other studies, Abed et al. showed a significant increase in the concentration of calcium in 15 samples of smokers' saliva compared to nonsmokers samples [8]. However, the major differences of Abed's study were including male subjects and unclear exclusion of study subjects which can possibly explain the different results.

Duration of smoking, type, and the number of smoked tobacco were related to the nicotine dose exposure. Longer smoking duration and higher number of smoked tobacco increase the exposure of nicotine to the oral cavity. Most kretek cigarettes have a higher level of nicotine compared to the other types of smoked tobacco [37]. In our study, we found that longer smoking duration, which conveys higher nicotine exposure, was associated with lower salivary calcium levels. Nicotine can reduce estrogen and parathyroid hormone (PTH) levels. These hormones affect salivary calcium levels [13, 38]. Estrogen is known to play a role in changing the composition of saliva. Salivary calcium levels increase when estrogen levels decrease. The decrease in estrogen in women who smoke can reduce calcium absorption in the small intestine by 20–25%, which in turn reduces blood calcium levels. Women who smoke also experience a decrease in PTH. This hormone has a role in balancing calcium levels in the blood by releasing calcium from the bones into the blood. PTH will increase when calcium in the blood decreases. But, in women who smoke, PTH does not work optimally. Blood calcium levels will remain reduced because of the lower calcium levels in saliva [39].

### 4.3. Bleeding on Probing (BOP)

We found that female smokers' mean BOP was higher than nonsmoker's. The majority of subjects in this study were not heavy smokers with an average cigarette consumed per day of fewer than ten cigarettes. Dietrich et al. found that smoking cigarettes causes a dose-dependent suppressive effect of gingival BOP [40]. In this study, there was a significant difference in BOP after adjusting for the duration of smoking. BOP decreases along with an increasing period of the smoking habit. Mean BOP of subjects who smoked more than ten cigarettes a day was also lower than that of control subjects.

The nicotine contained in cigarettes can stimulate the adrenal hormones. It causes the vasoconstriction of peripheral blood vessels and reduces the blood supply and oxygen to the gingiva. As a result, inflammatory and bleeding responses on probing in smokers are lower [10].

### 4.4. Calcium Intake

Mean calcium intake in both smokers and nonsmokers per day was inadequate. However, the mean calcium intake of female smokers was lower than that of nonsmokers. The optimal calcium intake value based on the National Institute of Health for women aged 19 years to menopause is 1000 mg/day [41].

The low percentage of calcium intake in both smokers and nonsmokers can be associated with the demographics of the study subjects [33]. The research subjects live in rural areas with low educational and occupational (economic status) backgrounds. After adjusting for smoking characteristics, the total calcium intake of female smokers still did not exhibit a significant difference in each category.

### 4.5. Correlation of Salivary Calcium Levels with Total Calcium Intake and BOP

We found that higher salivary calcium levels were correlated with higher BOP in female smokers, but this correlation was not found in nonsmokers. Previous studies investigating the correlation between salivary calcium levels and BOP in smokers and nonsmokers showed conflicting results. Sutej et al. reported that the increase of salivary calcium level was associated with the decrease in the BOP [42]. Kiss et al. found no significant relationship between salivary calcium levels and BOP in female smokers [43]. Meanwhile, Sewón et al. found that higher salivary calcium levels were associated with higher BOP in healthy subjects [44]. Plaque and calculus quality suggested being the reason for these differences [42, 44]. But, the subjects of the two groups in this study considered having generally poor oral hygiene due to not regularly visiting the dentist. Our findings suggest that the relationship between salivary calcium levels and BOP in smokers was affected by the dose of nicotine exposure. In further research, the measurements of plaque and calculus index might be required to find a profound understanding of the correlation between these variables.

This study found that the total calcium intake in female smokers and nonsmokers did not correlate with salivary calcium levels. An experimental study in female rats by Hattori et al. also found that lower calcium intake did not affect salivary calcium levels. The study reported that changes in calcium intake only affect urinary calcium levels. Meanwhile, the salivary calcium levels are controlled by the salivary glands through the mineral resorption system in the saliva [45].

## 5. Conclusions

A low level of education may be contributing to the smoking habit of subjects in this study. Salivary calcium levels were correlated with BOP in female smokers, which might be affected by the duration of smoking.

## Figures and Tables

**Table 1:** Basic characteristics of study subjects.

	Female smokers (*n* = 26)		Control (*n* = 37)		*p* value
	Total	Percentage (%)	Total	Percentage (%)	
Age (years)					≤0.001^*∗*^
18–35 (young adult)	4	15.4	27	73	
36–55 (middle aged)	21	80.8	10	27	
>55 (older adult)	1	3.8	0	0	
Body mass index (BMI)					0.005^*∗*^
<18.5	1	3.8	5	13.5	
18.5–24.9	5	19.2	19	51.4	
≥25	20	76.9	13	35.1	
Level of education					≤0.001^*∗*^
Primary	21	80.8	6	16.2	
Secondary	5	19.2	16	43.2	
Postsecondary	0	0	15	40.5	
Occupation					0.001^*∗*^
Housewife	20	76.9	11	29.7	
Civil servant	0	0.0	1	2.7	
Private employee	2	7.7	2	5.4	
Entrepreneur	4	15.4	2	5.4	
Professional	0	0	9	24.3	
Student	0	0	11	29.7	
Unemployed	0	0	1	2.7	

^*∗*^Statistically significant (*p* value <0.05) according to the chi-square test.

**Table 2:** Mean and standard deviations of salivary calcium levels, total calcium intake, and BOP in study subjects.

	Female smokers (*n* = 26)		Control (*n* = 37)		*p* value
	Mean	SD	Mean	SD	
Salivary calcium level (mmol/L)	1.16	0.93	1.80	0.96	0.002^*∗*^
Total calcium intake (mg)	391.69	181.1	500.47	429.38	0.748
BOP (%)	8.82	6.85	5.39	4.43	0.010^*∗*^

^*∗*^Statistically significant (*p* value <0.05) according to the Mann–Whitney *U* test.

**Table 3:** Salivary calcium levels, total calcium intake, and BOP based on smoking characteristic.

Characteristic	Salivary calcium level (mmol/L)		Total calcium intake (mg)		BOP (%)	
	Mean	SD	Mean	SD	Mean	SD
Duration of smoking						
<2 years (*n* = 3)	3.19	0.18	284.90	103.11	18.41	14.38
2–5 years (*n* = 5)	1.34	0.90	490.66	227.24	11.70	5.40
>5 years (*n* = 18)	0.78	0.43	382	172.73	6.42	3.63
*p* value	0.010^*∗*^		0.273		0.038^*∗*^	
Types of smoked tobacco product						
Kretek (*n* = 2)	2.81	0.28	510.35	444.11	27.20	11.03
Filtered kretek (*n* = 3)	0.97	0.68	393.60	25.62	4.74	4.36
Filtered white cigarettes (*n* = 21)	1.03	0.86	380.17	171.81	7.65	3.61
*p* value	0.156		0.934		0.035^*∗*^	
Number of smoked tobacco consumption/day						
<10 (*n* = 22)	1.28	0.96	409.59	174.42	9.49	7.15
≥10 (*n* = 4)	0.50	0.17	293.26	212.32	5.17	3.61
*p* value	0.055		0.256		0.201	

^*∗*^Statistically significant (*p* value <0.05) according to the Kruskal–Wallis test.

**Table 4:** Correlation of salivary calcium levels with total calcium intake and BOP.

The correlation of salivary calcium level with	Female smokers (*n* = 26)		Control (*n* = 37)	
	*r*	*p* value	*r*	*p* value
BOP (%)	0.481	0.020^*∗*^	−0.040	0.823
Calcium intake (mmol/L)	−0.058	0.793	−0.267	0.126

^*∗*^Statistically significant (*p* value < 0.05) according to the partial correlation (2-tailed).

## Data Availability

The datasets used and/or analyzed during the current study are available upon reasonable request.
